# Coronal Plane Alignment of the Knee (CPAK) distribution in a diverse Asian population: Influence of ethnicity, sex and bilaterality

**DOI:** 10.1002/jeo2.70192

**Published:** 2025-04-01

**Authors:** Azmi Rahman, Merrill Lee, Lenice Tan, Sabine Wong, Michael Saturnino, Glen Purnomo, Ming Han Lincoln Liow, Keng Jin Darren Tay, Hee Nee Pang, Seng Jin Yeo

**Affiliations:** ^1^ University of Oxford Oxford UK; ^2^ Singapore General Hospital Singapore; ^3^ National University of Singapore Singapore; ^4^ Philippine Orthopaedic Centre Manila Philippines; ^5^ St. Vincentius a Paulo Catholic Hospital Surabaya Indonesia; ^6^ National Hospital Surabaya Indonesia

**Keywords:** Asian, Chinese, Coronal Plane Alignment of Knee, CPAK, Indian, Malay, native alignment

## Abstract

**Purpose:**

In total knee arthroplasty (TKA), it remains unclear which patients benefit from correction versus restoration of native knee alignment. The Coronal Plane Alignment of the Knee (CPAK) classification system was introduced in 2021 to describe native alignment, helping to characterise the effect of different TKA alignment techniques. This study aims to describe CPAK in an ethnically diverse population and characterise the relationship between CPAK and ethnicity, as well as the bilaterality of osteoarthritis and other patient factors.

**Methods:**

503 primary TKAs were performed in a large tertiary institution in Singapore from 2014 to 2021. Pre‐operative anteroposterior knee radiographs were collected for 441 procedures—all had ethnicity, age, sex and body mass index data. The medial proximal tibial angle (MPTA) and lateral distal femoral angle (LDFA) were measured with good inter‐observer correlation. Knees were then classified into nine CPAK categories based on arithmetic hip–knee–ankle (aHKA) angle and joint line obliquity (JLO).

**Results:**

77% of the cohort were apex‐distal (CPAK 1, 2 and 3), and 59% were varus (CPAK 1, 4 and 7); 44% were CPAK 1 (varus + apex‐distal). Chinese and Indian knees followed near‐identical patterns: CPAK 1 (46%) > CPAK 2 (20%) > CPAK 4 (15%). Malay knees had significantly fewer CPAK 1 (*p* = 0.0183), with CPAK 1 (29%) ≈ CPAK 2 (29%) ≈ CPAK 4 (21%). Thirty‐eight patients had bilateral TKA. Identical categories were recorded bilaterally in 45% of CPAK, 67% of JLO and 70% of aHKA. Bilateral TKA were more likely when knees were in valgus alignment than unilateral TKA (*p* = 0.00457).

**Conclusion:**

Malay knees are less likely to be CPAK‐1; this novel finding may explain ethnic differences in TKA outcomes described in the literature. Less than half of the bilateral knees had the same CPAK category bilaterally. The implications of this bilateral CPAK incongruence are unclear and require further study.

**Level of Evidence:**

Level II, prospective cohort study.

**Abbreviations**
aHKA: arithmetic hip–knee–ankle
BMI: body mass index
CPAK: Coronal Plane Alignment of the Knee
HKA: hip–knee–ankle
JLO: joint‐line obliquity
LDFA: lateral distal femoral angle
MPTA: medical proximal tibial angle
OA: osteoarthritis
SD: standard deviation
TKA: total knee arthroplasty

## INTRODUCTION

Total knee arthroplasty (TKA) is the most common surgical management option for patients with end‐stage osteoarthritis (OA). Different implant alignment approaches have been proposed for TKA, such as mechanical and kinematic alignment, but existing literature does not consistently recommend any of the techniques as superior [2, 8, 11, 20]. An effective description of knee morphology is required to study the effect of these techniques across different patient morphologies. Until recently, there has been limited pragmatic characterisation of knee morphology in relation to implant alignment. In 2021, MacDessi et al. introduced the CPAK classification, which measures arithmetic hip–knee–ankle (aHKA) angle and joint‐line obliquity (JLO) from an antero‐posterior knee radiograph to classify knees to a 3 × 3 grid of based on constitutional limb alignment (varus, neutral and valgus) and JLO (apex distal, neutral and apex proximal), allowing patients to be classified as CPAK 1–9 [9]. Recent literature describing CPAK and outcomes has found links between CPAK and outcomes based on alignment types, indicating that CPAK holds potential in optimising TKA alignment [1, 18, 23].

Descriptive studies on CPAK distributions vary on CPAK distributions found in different countries. A systematic review of 6000 knees across Belgium, Taiwan, India, France and Japan identified the most common CPAK phenotype as CPAK‐1 or CPAK‐2 [12]. However, several of these studies do not describe patient ethnicity, and it is unclear which patient groups are included in the studies. While there have been efforts to describe knee anatomy in the South East Asian population [19], there has been no literature to date characterising ethnic CPAK differences in this multi‐ethnic population, which draws from East, South and Central Asia. In addition, there has been no description of CPAK where bilateral TKA has been clinically indicated, though it is generally assumed that this will be identical between knees. Therefore, the aim of this study is to characterise the relationship between CPAK and ethnicity, bilaterality of OA, and other patient factors.

## METHODS

Prospectively collected data from a longitudinally maintained institution registry at a tertiary orthopaedic centre in Singapore were retrospectively reviewed. Between 2014 and 2021, a total of 503 knee arthroplasties were performed in a single institution. The pre‐operative patient‐specific variables captured were ethnicity, age, sex, and body mass index (BMI). Patients not within the Chinese, Malay or Indian ethnicity categories [21] formed less than 1% of the cohort. Pre‐operative standing antero‐posterior long‐leg radiographs taken for clinical use by radiographers were collected for review. Radiographs were unavailable for 62 knees and excluded from the study, resulting in 441 knees assessed.

CPAK assessment was performed as outlined by MacDessi et al. [9]. The medial proximal tibial angle (MPTA) and lateral distal femoral angle (LDFA) were measured on anteroposterior radiographs for all patients pre‐operatively. The LDFA was measured as the angle between the mechanical axis of the femur (drawn along the mid‐diaphysis of the femur) and the distal femoral joint line (drawn between the distal points on the lateral and medial femoral condyles). The MPTA was measured as the medial angle between the mechanical axis of the tibia (drawn along the mid‐diaphysis of the tibia) and the proximal tibial joint line (drawn across the distal bony surfaces of the lateral and medial tibial plateaus). These values were used to calculate constitutional aHKA (MPTA − LDFA) and JLO (MPTA + LDFA). Patients were then classified into the nine CPAK groups based on their pre‐operative alignment, as outlined in Table 1.

**Table 1:** CPAK classification.

		aHKA (MPTA − LDFA)		
		Varus (aHKA <−2°)	Neutral (−2°<aHKA <2°)	Valgus (aHKA >2°)
JLO (MPTA + LDFA)	Apex distal (JLO < 177°)	CPAK 1	CPAK 2	CPAK 3
	Neutral (177° < JLO < 183°)	CPAK 4	CPAK 5	CPAK 6
	Apex proximal (JLO > 183°)	CPAK 7	CPAK 8	CPAK 9

Abbreviations: aHKA, arithmetic hip–knee–ankle; CPAK, Coronal Plane Alignment of the Knee; JLO, joint‐line obliquity; LDFA, lateral distal femoral angle; MPTA, medical proximal tibial angle.

CPAK classification via MPTA and LDFA measurement is reliable [9]. All radiographs were assessed by one observer, and 10% (n = 44) were randomly selected to be assessed by a second‐independent observer. Observers were medical professionals trained in CPAK measurement techniques. Intra‐class correlation (ICC) for mixed effects was performed to ensure method reliability.

### Statistics

CPAK distributions were analysed using frequency distributions and were visualised using histograms. Underlying aHKA and JLO measurements were visualised using scatterplots. Discrete categories were assessed with chi‐square tests where appropriate. Statistical significance is defined by *p* values of <0.05. Data were analysed and visualised using GraphPad Prism (GraphPad Software) and Excel (Microsoft). ICC assessment was performed on IBM SPSS Statistics for Windows, Version 25.0 (Armonk, N.Y., USA: IBM Corp. Released 2017).

### Ethics

This study was approved by the local institutional review board (SingHealth CIRB 2015/2632) and received the necessary patient consent for study and publication.

## RESULTS

### Baseline characteristics

The characteristics of the study cohort are outlined in Table 2.

**Table 2:** Baseline characteristics of cohort.

Characteristic	Value
Age	68.7 years (SD: 8.3)
BMI	27.6 (SD: 5.0)
Sex	Male: 119 (27%)
	Female: 322 (73%)
Side	Left: 204 (46%)
	Right: 237 (54%)
Ethnicity	Chinese: 384 (87%)
	Malay: 29 (7%)
	Indian: 23 (5%)
	Others: 5 (1%)

Abbreviations: BMI, body mass index; SD, standard deviation.

### Second observer assessment

LDFA and MPTA measurements were cumulatively assessed for method reliability. There was very good agreement between the main observer measurements and the second observer measurements (two‐way mixed‐effect ICC for absolute agreement: 0.841).

### CPAK distribution

**Figure 1:**
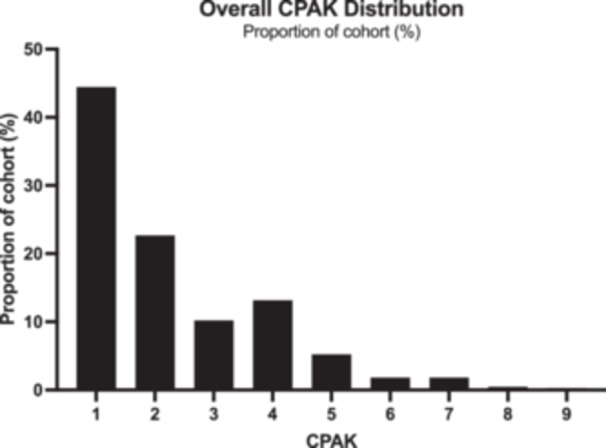
Histogram of overall CPAK distribution in cohort, represented as a proportion of cohort (%) per CPAK category.

Across the cohort, most of the arthritic knees were CPAK 1 (44%), followed by CPAK 2 (23%), CPAK 4 (13%), CPAK 3 (10%), CPAK 5 (5%), CPAK 6 (2%), and CPAK 7 (2%). <1% of the cohort were CPAK 8 or 9 (Figure 1).

**Figure 2:**
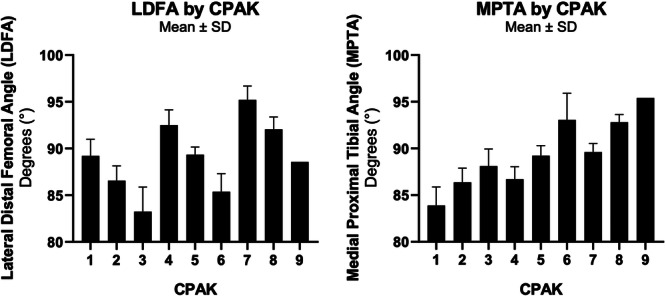
(a) Lateral distal femoral angle and (b) medial proximal tibial angle in each CPAK category. CPAK, Coronal Plane Alignment of the Knee; SD, standard deviation.

aHKA and JLO values varied with CPAK categories consistent with their definitions in the Methods section (Table 3). LFDA and MPTA both had significant differences across CPAK categories (ANOVA *p* < 0.0001 in both), generally increasing from CPAK 1 to 9 (Table 3, Figure 2a,b).

**Table 3:** Mean (SD) LDFA, MPTA, aHKA and JLO in each CPAK category.

CPAK category		LDFA	MPTA	aHKA	JLO
1	Mean	89.2	83.9	−5.31	173
	SD	1.78	1.99	2.83	2.49
2	Mean	86.6	86.4	−0.185	173
	SD	1.58	1.53	1.12	2.90
3	Mean	83.2	88.1	4.87	171
	SD	2.63	1.83	2.92	3.45
4	Mean	92.5	86.7	−5.79	179
	SD	1.63	1.32	2.49	1.61
5	Mean	89.3	89.2	−0.123	179
	SD	0.810	1.10	1.25	1.46
6	Mean	85.4	93.1	7.68	178
	SD	1.93	2.85	4.75	1.09
7	Mean	95.2	89.6	−5.60	185
	SD	1.48	0.930	1.83	1.66
8	Mean	92.1	92.8	0.745	185
	SD	1.32	0.827	0.488	2.14
9	Mean	88.6	95.4	6.85	184
	SD	0.00	0.00	0.00	0.00

Abbreviations: aHKA, arithmetic hip–knee–ankle; CPAK, Coronal Plane Alignment of the Knee; JLO, joint‐line obliquity; LDFA, lateral distal femoral angle; MPTA, medical proximal tibial angle; SD, standard deviation.

### CPAK by sex

**Figure 3:**
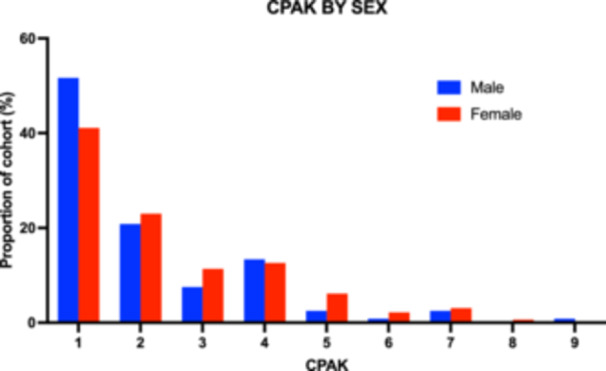
Histogram of CPAK distribution by male and female sex, represented as a proportion of cohort (%) per CPAK category. CPAK, Coronal Plane Alignment of the Knee.

There were small differences in CPAK distribution between male and female knee morphology—the largest difference noted was with 52% of male knees but 41% of female knees being CPAK 1. However, this difference was not statistically significant (chi‐square test for CPAK 1 vs. CPAK 2–9, *p* = 0.156) (Figure 3).

### CPAK by ethnicity

**Figure 4:**
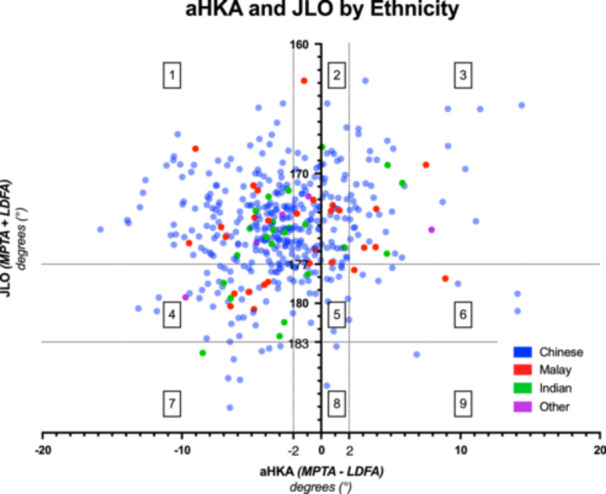
Scatterplot of arithmetic hip–knee–ankle (aHKA) angle against joint‐line obliquity (JLO), by ethnicity. CPAK thresholds are presented as lines with categories shown (CPAK 1–9, top left to bottom right). CPAK, Coronal Plane Alignment of the Knee; LDFA, lateral distal femoral angle; MPTA, medical proximal tibial angle.

The distribution of JLO and aHKA is presented in Figure 4.

**Figure 5:**
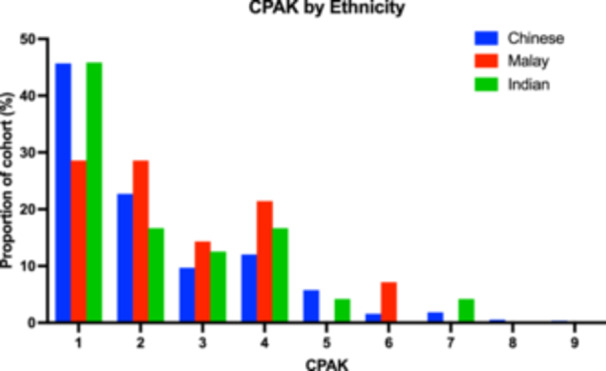
Histogram of CPAK distribution by Chinese, Malay and Indian ethnicity, represented as a proportion of cohort (%) per CPAK category. Other ethnicities were excluded from the figure due to small sample size (*n* = 5, <1% of cohort). CPAK, Coronal Plane Alignment of the Knee.

Chinese and Indian arthritic knees followed near‐identical patterns—CPAK 1 (Chinese 46%, Indian 46%), followed by CPAK 2 (Chinese 23%, Indian 17%), then CPAK 4 (Chinese 12%, Indian 17%). Malay knees had a different distribution, with significantly fewer CPAK 1 (chi‐square test for CPAK 1 vs. CPAK 2–9 across ethnicities, *p* = 0.0183), and a similar proportion of CPAK 1 (29%), CPAK 2 (29%) and CPAK 4 (21%) (Figure 5).

### CPAK in bilateral versus unilateral arthritis

**Figure 6:**
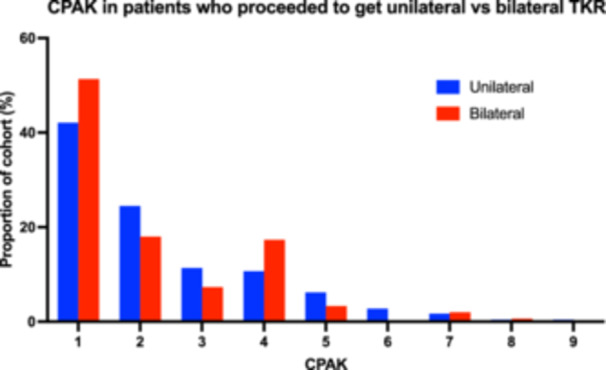
Histogram of CPAK distribution by patients who proceeded to get TKR on one (unilateral) or both (bilateral) TKR during the study period, represented as a proportion of cohort (%) per CPAK category. CPAK, Coronal Plane Alignment of the Knee; TKR, total knee replacement.

Arthritic knees were further classified between patients who proceeded to have total knee replacement (TKR) on both knees versus one side (Figure 6). Patients who had bilateral TKR were more likely to be genu varum (CPAK 1, 4 and 7), and this likelihood ratio increased from 1.2x for CPAK 1 (varus with apex distal) to 1.5x for CPAK 2 (varus with neutral)—individually, these differences were not statistically significant (chi‐square test for CPAK 1 vs. CPAK 2–9, *p* = 0.257; CPAK 4 vs. CPAK 1–3, 5–9, *p* = 0.310). However, when genu valgum morphologies were combined, there was a greater likelihood for bilateral TKR than unilateral TKR (chi‐square test for CPAK 1, 4, 7 vs. CPAK 2, 3, 5, 6, 8, 9, *p* = 0.00457).

### CPAK in patients undergoing bilateral OA

In patients with bilateral end‐stage osteoarthritic knees, 45% had the same CPAK on both sides, while 55% did not. In contrast, a larger 67% of patients with bilateral arthritis had the same JLO category (CPAK 1–3, CPAK 4–6, CPAK 7–9), and 70% had the same aHKA category (CPAK 1/4/7, CPAK 2/5/8, CPAK 3/6/9) (Table 4).

**Table 4:** CPAK, JLO and aHKA congruence in patients who had bilateral end‐stage osteoarthritis.

	Identical between sides (*n*, %)	Difference between sides (*n*, %)	
CPAK	34 (45)	42 (55)	76 (38 pairs)
JLO	51 (67)	25 (33)	
aHKA	53 (70)	23 (30)	

Abbreviations: aHKA, arithmetic hip–knee–ankle; CPAK, Coronal Plane Alignment of the Knee; JLO, joint‐line obliquity.

## DISCUSSION

The most notable finding of this study was that CPAK distributions of arthritic knees were significantly different between patients of Malay ethnicity and patients of the other ethnicities included in this study. CPAK distributions in Chinese and Indian ethnicities followed similar patterns of these populations described in other studies in Asian populations of similar ethnicities [4, 5, 10, 17, 22], with a majority of patients—nearly 50%—being CPAK 1, and the remaining patients being CPAK 2–9 in a progressively reducing incidence. This pattern is also found to be generally consistent with most CPAK distributions described in North American and European studies [3, 9, 14].

This study demonstrates patients of Malay ethnicity present with a significantly lower likelihood of being CPAK 1 compared to other ethnicities (29% vs. 46%), compensated with a greater incidence in CPAK 2, 4, 6. A study in a similar population analysed the relationship between ethnicity and CPAK alignment parameters (aHKA, JLO, LDFA and MPTA) and did not find significant differences between groups; however, it did not compare overall CPAK categories between ethnicities [19]. This CPAK difference demonstrates that the Malay population included in this study is generally more likely to have a neutral (instead of valgus) aHKA and a neutral (instead of apex distal) JLO. There is limited evidence directly implicating this as a factor worsening patient outcomes. However, it is shown that patients with Malay ethnicity have poorer patient‐reported pain and function outcomes [13, 16]. These may be related to suboptimal implant alignment and require further ethnicity‐matched analysis of CPAK and its association with outcomes.

Second, this study newly describes the likelihood of congruence of CPAK and underlying HKA and JLO groups between bilateral arthritic knees. In the subset of patients included in this study where both knees were assessed, less than half of them (45%) were determined to have the same CPAK category. This incongruence in CPAK appears to be contributed equally by incongruence in aHKA (67% congruence) and JLO (70% congruence). CPAK congruence between arthritic knees has not been described before. It is generally assumed that knee alignment is identical bilaterally, without trauma, underlying deformity, or other pathology. However, this study found small but consistent differences were seen between CPAK distributions and patients who underwent bilateral TKR in the cohort. Differences were linked to underlying valgus HKA groups (CPAK 3, 6 and 9), which is a known risk factor [6, 15]. It is unclear why this difference is observed—this relationship requires further study to determine if differences are clinically relevant to patient outcomes or present a difference in classification without clinical effect.

In addition to ethnic and bilaterality differences, this study also found that males tended to be slightly more likely to be CPAK 1 than females. However, this difference was not statistically significant. Larger studies in the literature have identified similar patterns with larger cohorts [7], suggesting that this study is likely underpowered to conclusively determine the relationship. Further study with larger cohorts and with ethnicity subsets is needed.

It is noted that patient ethnicity used in this study was based on patient‐reported identification data, which may, though unlikely, be inaccurate. Patients were required to identify with a single main ethnicity, with mixed ethnicities not accounted for. Patients identifying as Chinese are relatively over‐represented in the cohort compared to the population baseline. Assessment of bilaterality was limited due to a small sample size. In addition, patient inclusion depended on clinical assessment for end‐stage OA and subsequent TKR; this would have happened at different time points for both left and right knees. However, it is highly unlikely that a chronological change in CPAK trends will occur over time, and therefore, this is unlikely to have introduced any bias to the study.

## CONCLUSION

In the Southeast Asian population studied, Malay knees are less likely to be CPAK 1 than other ethnicities; this novel finding may explain ethnic differences in TKA outcomes described in the literature. Less than half of the bilateral knees had the same CPAK category bilaterally. While it is unlikely to have clinical implications, the implications of this bilateral CPAK incongruence are unclear, and further study may offer insight into CPAK classification.

## AUTHOR CONTRIBUTIONS

All authors contributed substantially to the conception or development of the study, data collection, data analysis and preparation of the manuscript.

## CONFLICT OF INTEREST STATEMENT

The authors declare no conflicts of interest.

## ETHICS STATEMENT

This study was approved by the local institutional review board (SingHealth CIRB 2015/2632) and received the necessary patient consent for study and publication.

## Data Availability

The data that support the findings of this study are available from the corresponding author upon reasonable request.
